# Grandmother’s Diet Matters: Early Life Programming with Sucrose Influences Metabolic and Lipid Parameters in Second Generation of Rats

**DOI:** 10.3390/nu12030846

**Published:** 2020-03-21

**Authors:** Elena Školníková, Lucie Šedová, Ondřej Šeda

**Affiliations:** Institute of Biology and Medical Genetics, First Faculty of Medicine, Charles University and the General University Hospital, 128 00 Prague, Czech Republic; elena.skolnikova@gmail.com (E.Š.); lsedo@lf1.cuni.cz (L.Š.)

**Keywords:** high sucrose diet, rat model, DOHAD, HDL cholesterol, brown fat

## Abstract

Early life exposure to certain environmental stimuli is related to the development of alternative phenotypes in mammals. A number of these phenotypes are related to an increased risk of disease later in life, creating a massive healthcare burden. With recent focus on the determination of underlying causes of common metabolic disorders, parental nutrition is of great interest, mainly due to a global shift towards a Western-type diet. Recent studies focusing on the increase of food or macronutrient intake don’t always consider the source of these nutrients as an important factor. In our study, we concentrate on the effects of high-sucrose diet, which provides carbohydrates in form of sucrose as opposed to starch in standard diet, fed in pregnancy and lactation in two subsequent generations of spontaneously hypertensive rats (SHR) and congenic SHR-Zbtb16 rats. Maternal sucrose intake increased fasting glycaemia in SHR female offspring in adulthood and increased their chow consumption in gravidity. High-sucrose diet fed to the maternal grandmother increased brown fat weight and HDL cholesterol levels in adult male offspring of both strains, i.e., the grandsons. Fasting glycaemia was however decreased only in SHR offspring. In conclusion, we show the second-generation effects of maternal exposition to a high-sucrose diet, some modulated to a certain extent by variation in the *Zbtb16* gene.

## 1. Introduction

The early development of a mammalian organism represents a critical time in the determination of health and disease in its adult life. During this period, a specific time windows occur, when the developing organism is particularly susceptible to environmental exposures capable of changing its phenotype outcome. Nutrition represents one of the most important factors that influence the highly sensitive developing fetus and does so through a complex of maternal physiology and the uteroplacental unit [1].

The availability and composition of nutrients affect epigenetic marks as they are being established in the developing organism, affect its development through changes in activity of its genes and by modulating metabolic pathways. Diet as well as other environmental factors influence the establishment or maintenance of specific epigenetic patterns through the activity of methyltransferases [2] or histone modifications [3,4]. When it comes to DNA methylation, not only do the activity of methyltransferases matter. The substrate–methyl donors also must be provided by the maternal diet [5]. It’s important to mention that epigenetic modifications can be maintained after mitotic cell divisions [6,7] and without regard to established pattern, the action of DNMT1 (DNA methyltransferase 1) is responsible for maintenance of these methylation patterns throughout life. That is why early life exposure to environmental stimuli with the capacity to permanently alter epigenome can impact health/disease status later in life [6,7]. On the other hand, the importance of the genomic background of both mother and fetus for the risk of adult disease as well as for the sensitivity to environmental stimuli has also been repeatedly shown [8,9].

Truly transgenerational transfer can however only occur if none of the cell lines are directly influenced by the environmental stimulus. If F0 generation female is influenced by a stimulus during pregnancy, the F2 generation germline in developing F1 fetus is affected, and therefore observed effect is usually denoted as intergenerational [10,11].

Due to the raised interest in environmental factors able to alter the mammalian development towards disease in adult life (DOHAD, developmental origins of health and disease), many studies using animal models have focused on understanding the underlying mechanisms in past years. The effects of micronutrient intake and various dietary regimens of parents on postnatal health of the offspring have been extensively reported [12,13,14,15,16,17]. Although many studies focus on the pressing problem of parental overnutrition and how it influences the progeny, the role of individual sources of macronutrients in these effects is yet to be understood. Our study takes a closer look at how the source of carbohydrates (starch versus sucrose) in the diet matters, even though it provides a comparable amount of calories.

In this paper, we present the effects apparent in two subsequent generations of offspring of two genetically distinct rat models, focusing on the second generation (F2). In addition to alternative carbohydrate source in maternal diet we introduced a small genetic difference in Zbtb16 (Zbtb16—Zinc Finger and BTB Domain Containing 16) gene between animals in the study design. Recent studies show that Zbtb16 acts as a pleiotropic factor involved in embryogenesis, cell differentiation and lipid and glucose metabolism [18]. We and others previously established the importance of *Zbtb16* gene in metabolic syndrome and its nutrigenetic aspects [18,19,20]. In particular, *Zbtb16* targeted models of mice [19] and rats [20] show significantly reduced levels of triacylglycerols and cholesterol and exhibited lower levels of serum insulin and significantly increased sensitivity of adipose and muscle tissue to insulin action, respectively. Therefore, we also tested the hypothesis that genetic variation in *Zbtb16* will affect the potential intergenerational effects of sucrose feeding. Since the genetic difference between the two used strains is only subtle, we opted to enhance homogeneity of the experimental groups by using highly inbred strains of animals and limiting our focus only to male F2 offspring of F1 programmed rat dams.

## 2. Materials and Methods 

Experiments were performed in agreement with the Animal Protection Law of the Czech Republic (311/1997) which is in compliance with the European Community Council recommendations for the use of laboratory animals 86/609/ECC and were approved by the Ethical Committee of the First Faculty of Medicine of the Charles University and by the Ministry of Education, Youth and Sports (protocol no. MSMT-14076/2015-14).

We used the spontaneously hypertensive rat (SHR/OlaIpcv, RGD ID 631848) because of its inherent abnormal metabolic profile [21] and its sensitivity manipulation by diet [22]. The SHR-Lx.PD5^PD-*Zbtb16*^ single congenic strain (SHR-Zbtb16 hereafter) was derived from polydactylous rat (PD/Cub, RGD ID 728161) [23] and carries the *Zbtb16* gene of PD origin on the SHR genomic background [24,25]. Both strains are highly inbred and maintained by brother x sister mating at the Institute of Biology and Medical Genetics.

**Figure 1:**
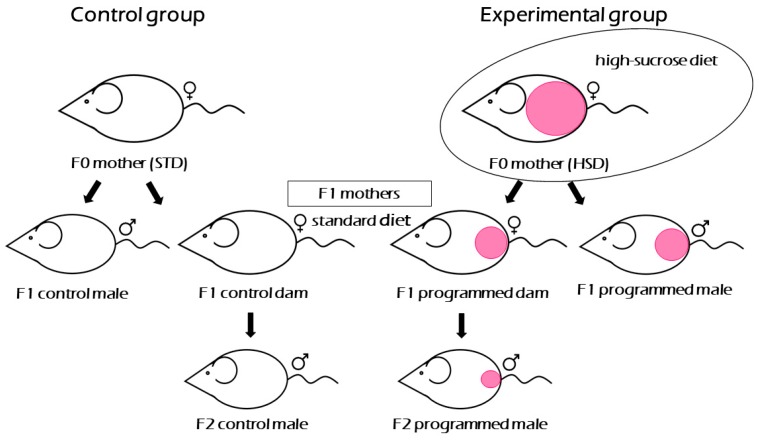
Experimental design. Schematic display of three generations of rats involved in the experiment valid for both SHR and SHR-Zbtb16 strain. Only F0 mothers of the experimental group were fed high-sucrose diet. Current study focuses on F1 generation female groups mothering F2 generation males. STD: standard diet, HSD: high-sucrose diet.

All animals were held under controlled conditions (temperature, humidity) with free access to food and water. F0 generation of rat dams (grandmothers in this study) of SHR and SHR-Zbtb16 strains (*n* = 6/strain) came from standard breeding and were fed a standard diet till the age of 16 weeks when they entered the experimental protocol. To recapitulate, F0 rat dams were fed standard diet till breeding with corresponding (SHR × SHR, SHR-Zbtb16 × SHR-Zbtb16) males fed standard diet. After mating, rat dams were placed in the cages individually and fed either standard diet (STD, ssniff Rat breeding V1324-000, ssniff Spezialdiäten GmbH, Soest, Germany) in control group or high-sucrose diet (HSD, proteins (19.6 cal%), fat (10.4 cal%), carbohydrates (sucrose, 70 cal%) [23] prepared by Institute for Clinical and Experimental Medicine, Prague, Czech Republic) in experimental group (Figure 1). The diets differed in the carbohydrate fraction only, with starch in STD vs. sucrose in HSD as a source of carbohydrates; otherwise they contained equal amounts of macro- and micronutrients. Each group was fed either STD or HSD throughout pregnancy and lactation. The litter size was restricted to 8 pups both in SHR and SHR-Zbtb16 offspring which were weaned after 28 days and fed standard diet till adulthood. Female offspring of F0 generation were fed standard diet till the age of 4 months and used as F1 generation (mothers in this study) in the same breeding model as their mothers.

F1 rat dams (*n* = 7/strain) were weighed regularly and subjected to OGTT at 16 weeks of age to determine the differences in glucose tolerance after being metabolically programmed by their maternal diet—either STD or HSD. Blood samples for metabolic and glycemic assessments were drawn after overnight fasting from the tail vein. The blood samples were obtained at intervals of 0, 30, 60, 120, and 180 min after intragastric glucose administration to conscious rats (3 g/kg body weight, 30% aqueous solution; Ascensia Elite Blood Glucose Meter; Bayer HealthCare, Mishawaka, IN, USA; validated by the Institute of Clinical Biochemistry and Laboratory Diagnostics of the First Faculty of Medicine). After the assessment of their adult glucose tolerance, the rat dams were mated with corresponding STD-fed males ((SHR x SHR, SHR-Zbtb16 x SHR-Zbtb16), not programmed, outside the experiment) and placed individually in cages. We assessed their weight gain, amount of chow consumed and again their glucose tolerance on the 10th day of pregnancy. Each group of F1 rat dams was fed standard diet whole 3 weeks of pregnancy and 4 weeks of lactation. The litter size was restricted to 8 pups both in SHR and SHR-Zbtb16 offspring which were weaned after 28 days and fed STD till adult age of 6 months creating the F2 generation. At that time, SHR and SHR-Zbtb16 male offspring of both control (*n* = 8/strain) and experimental (*n* = 8/strain) groups were subjected to OGTT, blood draw for metabolic and lipid profile assessment and sacrificed to determine the weights of heart, liver, kidneys, adrenals, interscapular brown fat, epididymal and retroperitoneal fat pads. The lipid profile was assessed using high performance liquid chromatography (HPLC) for determining triglyceride (TG) and cholesterol (C) concentrations in 20 lipoprotein fractions and the size of major classes of lipoprotein particles as described previously [25,26].

All statistical analyses were performed using STATISTICA 13.3 (TIBCO Software Inc.). When comparing morphometric and biochemical variables between groups, two-way ANOVA with STRAIN and DIET as major factors were used, followed by post-hoc Fisher’s test for comparison of the specific pairs of variables. Null hypothesis was rejected whenever *p* > 0.05.

## 3. Results

### 3.1. F1 Female Rats—Mothers

**Figure 2:**
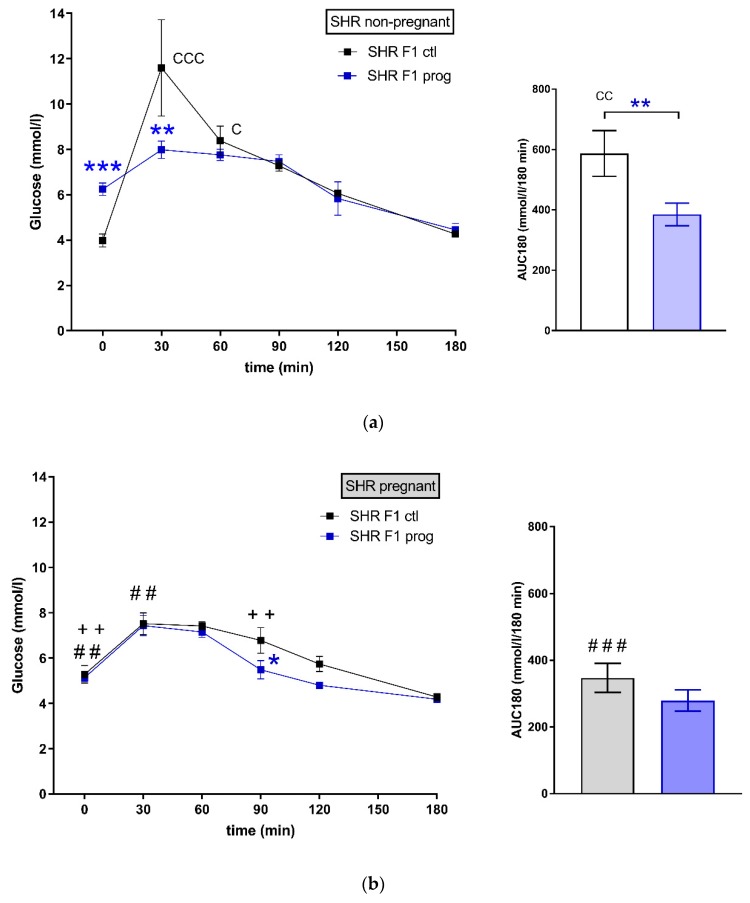

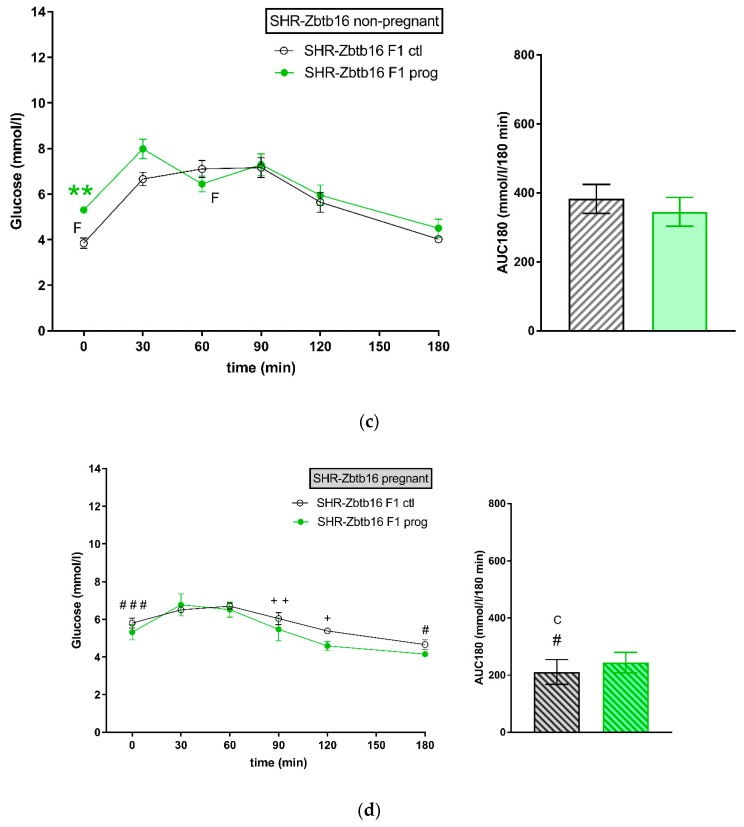
The oral glucose tolerance test (OGTT). The course of glycaemic curves in rat dams in adulthood one week prior (**a**,**c**) and during pregnancy (**b**,**d**); SHR F1 controls—black boxes, F1 programmed SHR—blue boxes, SHR-Zbtb16 F1 controls—white circles, F1 programmed SHR-Zbtb16—green circles. Data are expressed as mean ± SEM. The strain comparison using the post-hoc Fisher’s least significant difference test of the two-way ANOVA for STRAIN and PROGRAM as major factors are indicated as follows: * *p* < 0.05, ** *p* < 0.01, *** *p* < 0.001. Effect of programming in SHR and SHR-Zbtb16 strains is represented by blue and green asterisks (*), respectively. (**a**) C represents STRAIN differences between SHR and SHR-Zbtb16 F1 controls in adulthood before the pregnancy, t = 30 min CCC *p* < 0.001 , t = 60 min C *p* < 0.05 , AUC180 CC *p* < 0.01, which were all higher in SHR; (**b**) # represents differences in non-pregnant and pregnant SHR F1 controls (effect of pregnancy), t = 0 min ## *p* < 0.01, higher in pregnant SHR F1 controls, t = 30 min ##, AUC180 ### *p* < 0.001 , higher in non-pregnant SHR F1 controls, + represents differences in non-pregnant and pregnant SHR F1-programmed dams (effect of pregnancy), t = 0 min ++ *p* < 0.01 , t = 90 min ++, which were all higher in non-pregnant SHR F1-programmed dams; (**c**) F represents strain differences between SHR and SHR-Zbtb16 F1-programmed dams in adulthood before the pregnancy, t = 0 min *, t = 60 min *, which were both higher in SHR F1-programmed dams; (**d**) C represents STRAIN differences between SHR and SHR-Zbtb16 F1 pregnant controls, AUC180 C *p* < 0.05, which was higher in SHR F1 controls, # represents differences in non-pregnant and pregnant SHR-Zbtb16 F1 controls (effect of pregnancy), t = 0 min ### *p* < 0.001, t = 180 min # *p* < 0.05, higher in pregnant SHR-Zbtb16 F1 controls, AUC180 # *p* < 0.05, higher in non-pregnant SHR-Zbtb16 F1 controls, + represents differences in non-pregnant and pregnant SHR-Zbtb16 F1-programmed dams (effect of pregnancy), t = 90 min ++ *p* < 0.01, t = 120 min + *p* < 0.05, which were all higher in non-pregnant SHR-Zbtb16 F1-programmed dams.

**Figure 3:**
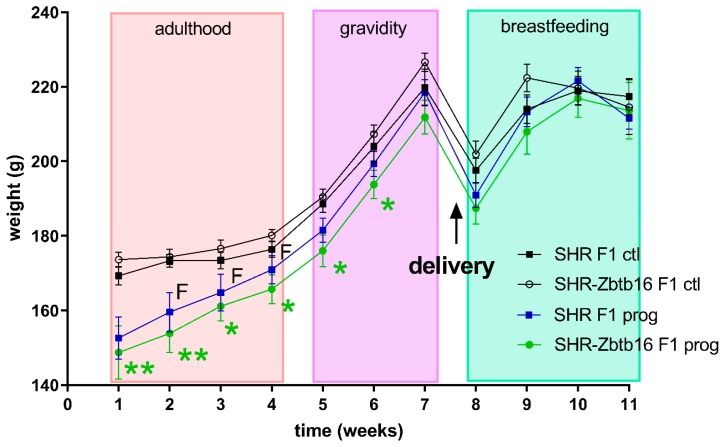
Body weight measurements of F1 SHR and SHR-Zbtb16 adult female rats during weeks prior to pregnancy (weeks 1–4), in pregnancy (weeks 5–7) and during lactation (weeks 8–11). Delivery occurred between weeks 7 and 8. SHR F1 controls—black boxes, F1-programmed SHR—blue boxes, SHR-Zbtb16 F1 controls—white circles, F1-programmed SHR-Zbtb16—green circles. Data are expressed as mean ± SEM. The strain comparison using the post-hoc Fisher’s least significant difference test of the two-way ANOVA for STRAIN and PROGRAM as major factors are indicated as follows: * *p* < 0.05, ** *p* < 0.01. Effect of programming in SHR and SHR-Zbtb16 strains is represented by blue and green asterisks (*), respectively. F represents strain differences between F1-programmed SHR and SHR-Zbtb16 dams, week 2 *, week 3 *, week 4 *, which were all higher in SHR F1-programmed dams.

There was a strain difference between control groups of female SHR F1 (Figure 2a) and SHR-Zbtb16 F1 rat dams (Figure 2c) in glucose tolerance prior to pregnancy (corresponding to week 4 in Figure 3). The difference in glucose tolerance between the strains diminished during pregnancy (Figure 2b,d).

**Figure 4:**
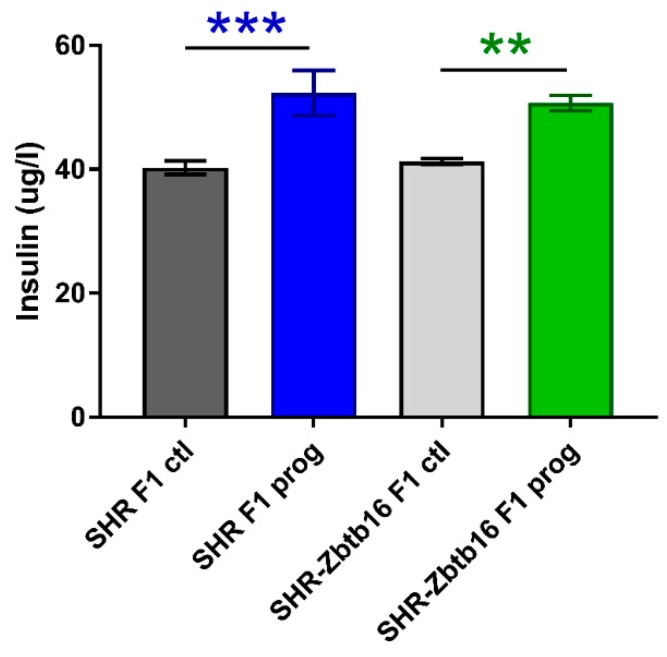
Fasting insulin concentrations in SHR F1 control females (dark grey bars), SHR-Zbtb16 F1 control females (light grey bars), F1-programmed SHR females (blue bars), F1-programmed SHR-Zbtb16 females (green bars) in adulthood. The strain comparison using the post-hoc Fisher’s least significant difference test of the two-way ANOVA for STRAIN and PROGRAM as major factors are indicated as follows: ** *p* < 0.01, *** *p* < 0.001. Effect of programming in SHR and SHR-Zbtb16 strains is represented by blue and green asterisks (*), respectively.

Both groups of rat dams programmed with maternal HSD exhibited significantly higher levels of fasting blood glucose compared to the control groups. The effect of pregnancy itself resulted in smaller area under the glycaemic curve in HSD-programmed groups (Table 1), as their response to glucose load had decreased. HSD-programmed females of both strains showed higher levels of fasting plasma insulin prior to pregnancy in comparison to control groups (Figure 4).

**Figure 5:**
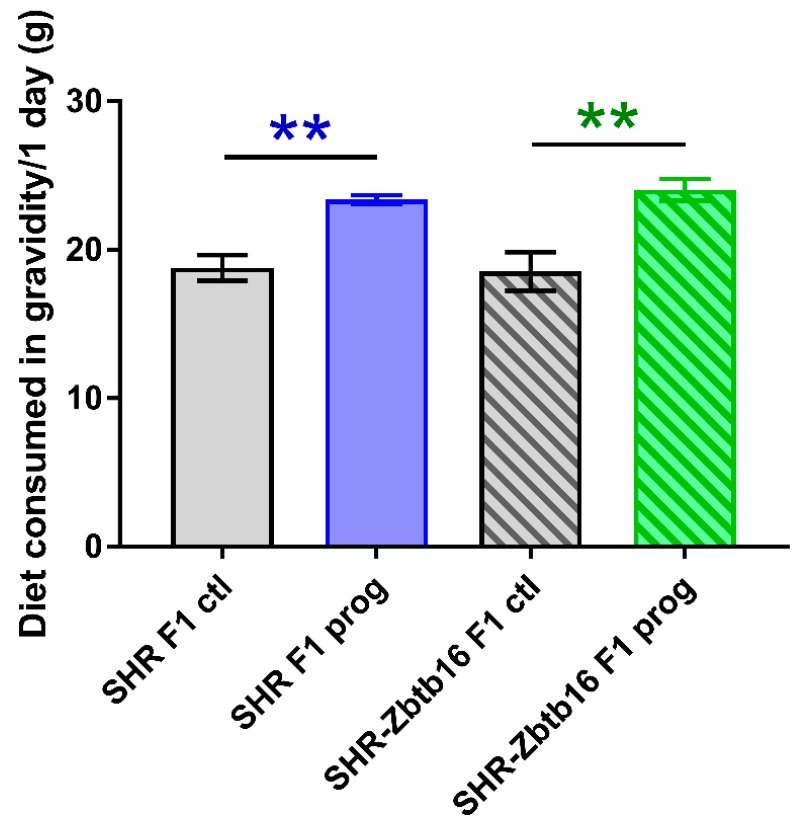
Diet consumption of F1 SHR and SHR-Zbtb16 adult female rats in gravidity. SHR F1 control females (grey bars), SHR-Zbtb16 F1 control females (grey patterned bars), F1-programmed SHR females (blue bars), F1-programmed SHR-Zbtb16 females (green patterned bars) daily consumption throughout 3 weeks of gravidity. Data are expressed as mean ± SEM. The strain comparison using the post-hoc Fisher’s least significant difference test of the two-way ANOVA for STRAIN and PROGRAM as major factors are indicated as follows: ** *p* < 0.01. Effect of programming in SHR and SHR-Zbtb16 strains is represented by blue and green asterisks (*), respectively.

As denoted in Figure 2, we observed several significant differences in oral glucose tolerance test (OGTT) data between strains prior or during the pregnancy with glycaemia being higher in SHR compared to SHR-Zbtb16. Control groups of female rats of both strains showed similar weight during whole 11-week period of measurement, as opposed to HSD -programmed females showing slight strain differences prior to gravidity (Figure 3; body weight 3 weeks before pregnancy 160 ± 5 g in SHR vs. 154 ± 5 g in SHR-Zbtb16, *p* = 0.046; body weight 2 weeks before pregnancy, 165±5 g in SHR vs. 161±4 in SHR-Zbtb16, *p* = 0.033; body weight 1 week before pregnancy 171 ± 4 g in SHR vs. 166 ± 4 g in SHR-Zbtb16, *p* = 0.03) as SHR dams were heavier. The most evident trend was the body weight difference between adult control and HSD-programmed F1 SHR-Zbtb16 females at the same age, reaching as far as the second week of pregnancy, when the difference became nonsignificant and remained so until the period after delivery (Figure 3). Interestingly, HSD-programmed rat dams of both strains ingested significantly larger amounts of chow during pregnancy and thus increased their caloric intake compared to control groups (Figure 5).

### 3.2. F2 Adult Male Offspring

**Figure 6:**
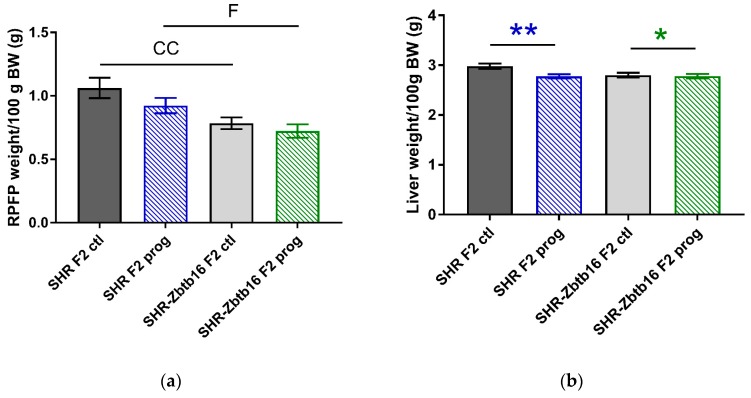

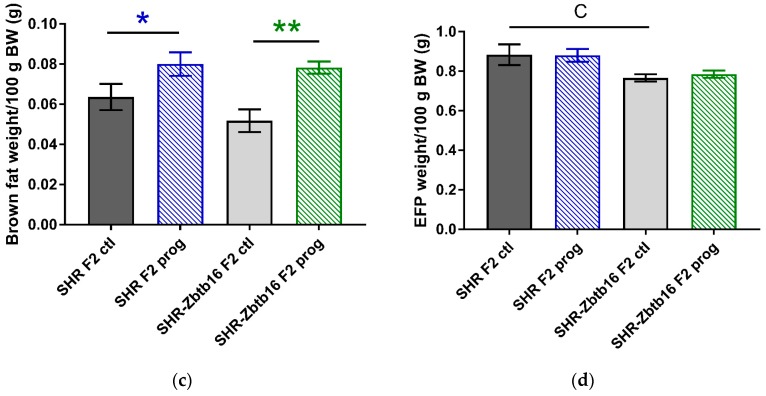
Morphometric assessment. Retroperitoneal fat pad weight (**a**), liver weight (**b**), interscapular brown fat weight (**c**), epidydimal fat pad weight (**d**) per 100 g of body weight in adult SHR F2 control male offspring (dark grey bars), SHR-Zbtb16 F2 control male offspring (light grey bars), F2-programmed SHR male offspring (blue patterned bars), F2-programmed SHR-Zbtb16 male offspring (green patterned bars) in 6 months of age. The strain comparison using the post-hoc Fisher’s least significant difference test of the two-way ANOVA for STRAIN and PROGRAM as major factors are indicated as follows: * *p* < 0.05, ** *p* < 0.01. Effect of programming in SHR and SHR-Zbtb16 strains is represented by blue and green asterisks (*), respectively. C represents STRAIN differences between F2 SHR and SHR-Zbtb16 control males, RPFP/100g BW CC *p* < 0.01, higher in SHR F2 control males; EFP/100 g BW C *p* < 0.05, higher in SHR F2 control males. F represents STRAIN differences between F2-programmed SHR and SHR-Zbtb16 males, RPFP/100 g BW *, higher in SHR F2-programmed males.

After delivery we didn’t observe any significant differences in litter size or birth weight of the pups among the groups. Adult F2 male offspring of HSD—programmed mothers did not differ in body weight from control animals, however, SHR were heavier than SHR-Zbtb16, similar to their mothers (body weight, 312 ± 7 g in SHR F2 controls vs. 298 ± 3 g in SHR-Zbtb16 F2 controls, *p* = 0.01; 321 ± 3 g in SHR F2 programmed males vs. 301 ± 5 g in SHR-Zbtb16 F2 programmed males, *p* = 0.03). Morphometric analysis of the animals revealed significantly decreased liver mass/100g of body weight in F2 programmed animals with more prominent effect in SHR males (Figure 6b). Second-generation programming affected body fat distribution with increased weight of interscapular brown fat pad (Figure 6c) compared to respective control groups. While the F2 control SHR-Zbtb16 male offspring had lower weight of epididymal fat/100 g of body weight than F2 control SHR offspring, there was no difference between the animals from the F2 programmed groups (Figure 6d).

**Figure 7:**
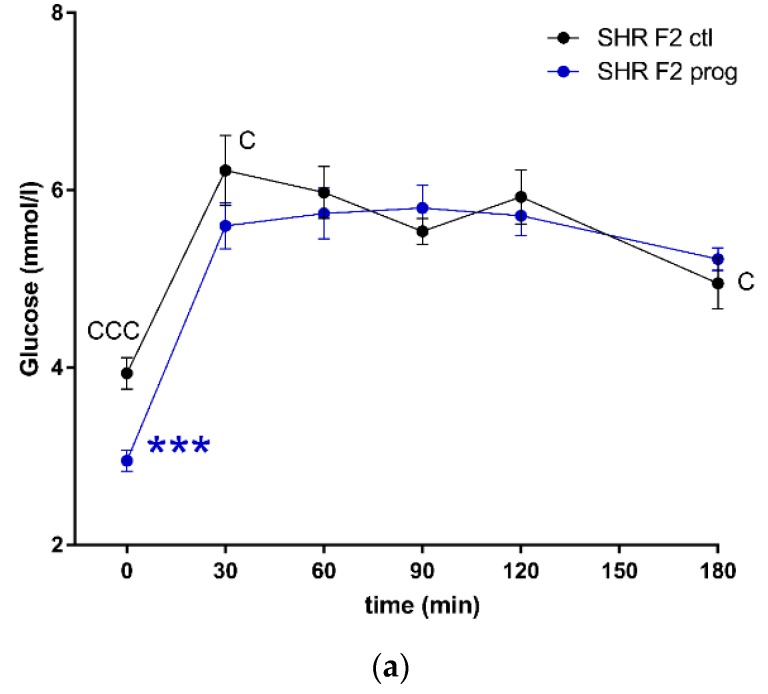

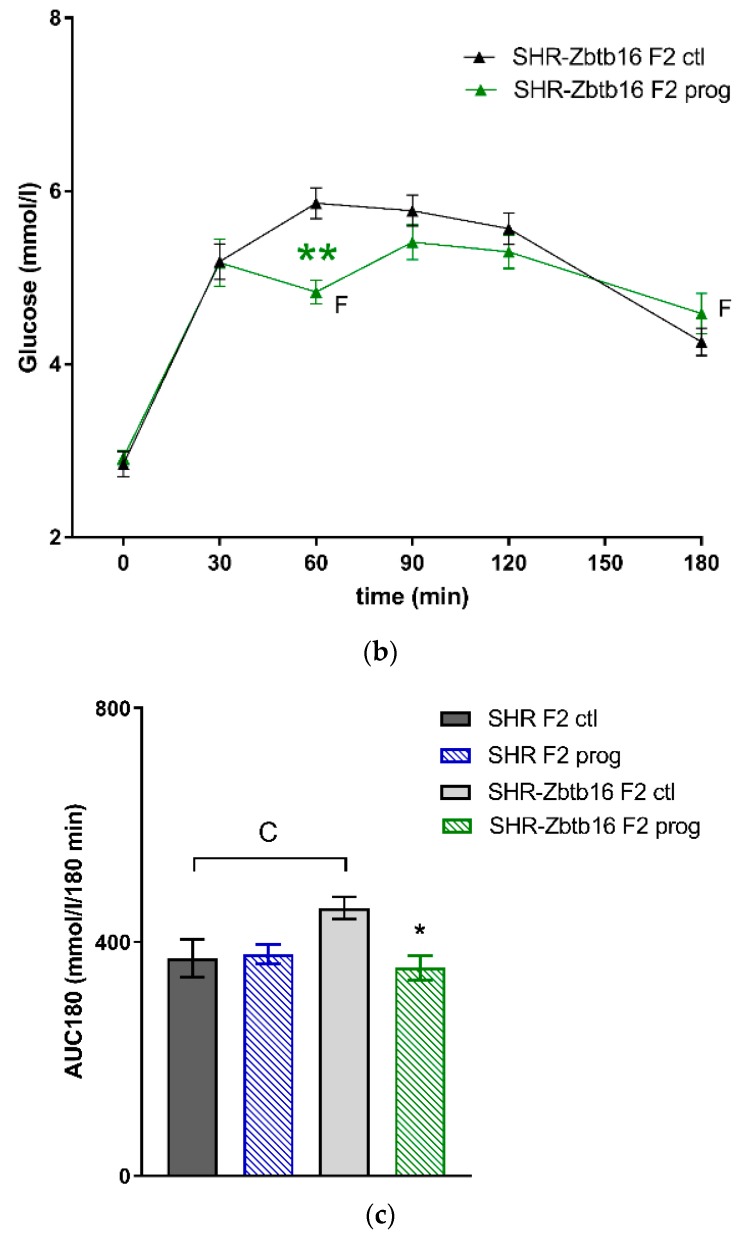
The oral glucose tolerance test (OGTT). The course of glycaemic curves in SHR F2 male offspring (a, control – black circles, programmed – blue circles), SHR-Zbtb16 F2 male offspring (b, control – black triangles, programmed – green triangles) during the oral glucose tolerance test and the corresponding areas under the glycaemic curves (c, AUC180). Data are expressed as mean ± SEM. The strain comparison using the post-hoc Fisher’s least significant difference test of the two-way ANOVA for STRAIN and PROGRAM as major factors are indicated as follows: * *p* < 0.05, ** *p* < 0.01, *** *p* < 0.001. Effect of programming in SHR and SHR-Zbtb16 strains is represented by blue and green asterisks (*), respectively. (**a**) C represents STRAIN differences between F2 SHR and SHR-Zbtb16 control males, t = 0 min CCC *p* < 0.001, t = 30 min C *p* < 0.05, t = 180 min C, which were all higher in SHR F2 control males. (**b**) F represents STRAIN differences between F2-programmed SHR and SHR-Zbtb16 males, t = 60 min *, t = 180 min *, which were both higher in SHR F2-programmed males; (**c**) C represents STRAIN differences between F2 SHR and SHR-Zbtb16 control males, AUC180 C *p* < 0.05, higher in SHR-Zbtb16 F2 control males.

**Figure 8:**
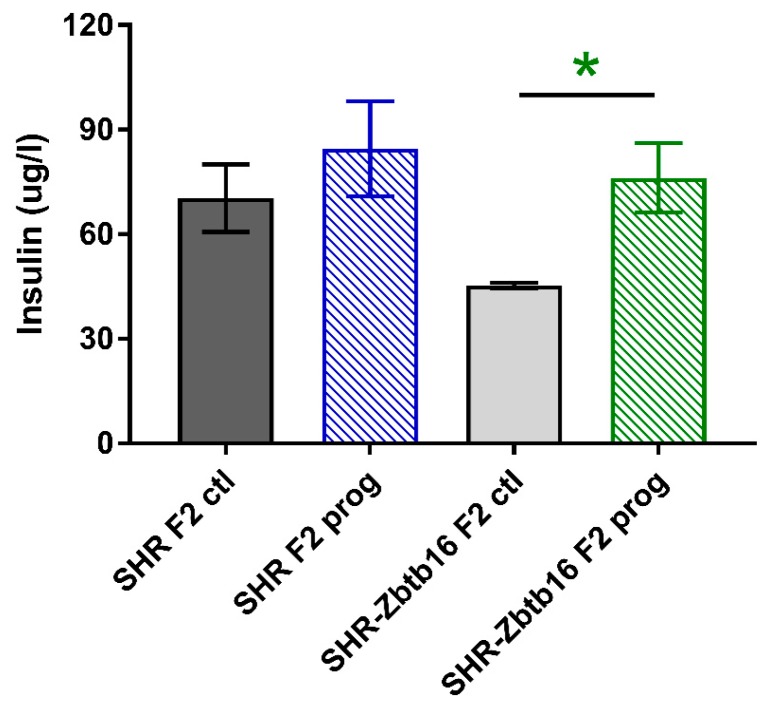
Fasting insulin concentrations in adult SHR F2 control male offspring (dark grey bars), SHR-Zbtb16 F2 control male offspring (light grey bars), F2-programmed SHR male offspring (blue patterned bars), F2-programmed SHR-Zbtb16 male offspring (green patterned bars) in 6 months of age. The strain comparison using the post-hoc Fisher’s least significant difference test of the two-way ANOVA for STRAIN and PROGRAM as major factors are indicated as follows: * *p* < 0.05. Effect of programming in SHR-Zbtb16 strain is represented by green asterisks (*).

F2 programmed SHR males displayed lower fasting glycaemia compared to their control group (Figure 7a), with no corresponding difference present in SHR-Zbtb16. F2 programmed SHR-Zbtb16 males displayed a biphasic OGTT like their F1 programmed mothers (Figure 7b), although AUC was not significantly different between the groups (Figure 7c, Table 1). In both control and programmed groups, we observed higher glycemia in SHR compared to SHR-Zbtb16 three hours after the glucose bolus. Fasting plasma insulin concentrations of adult offspring showed no difference between the control groups and a strain-specific increase of insulin levels in F2 programmed group of SHR-Zbtb16 (Figure 8).

**Figure 9:**
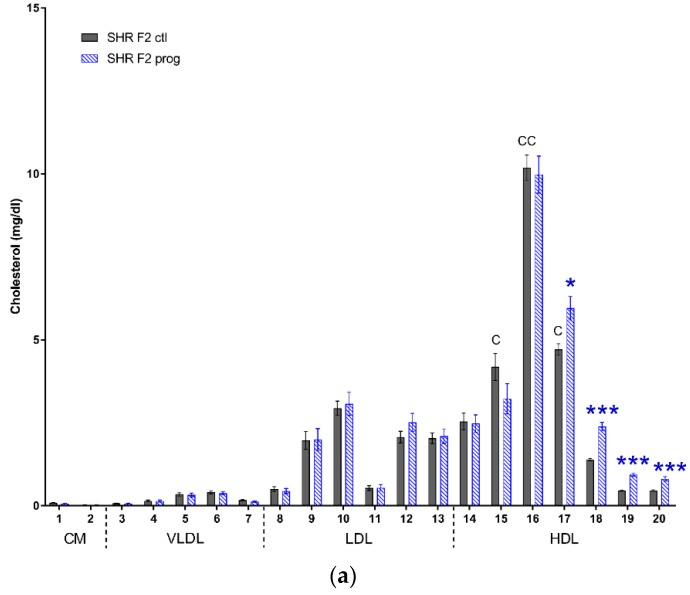

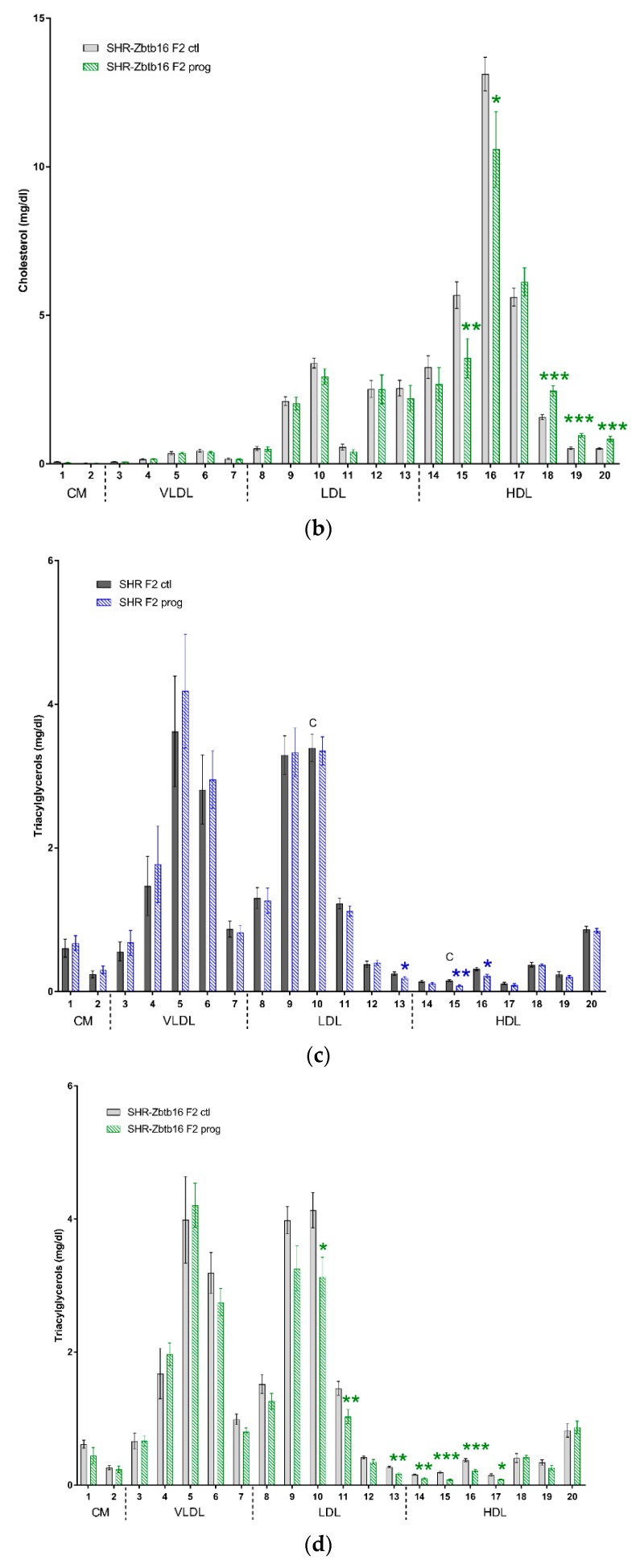
Lipoprotein profile. The cholesterol (**a**,**b**) and triacylglycerol (**c**,**d**) content in 20 lipoprotein subfractions in adult SHR F2 control male offspring (dark grey bars), SHR-Zbtb16 F2 control male offspring (light grey bars), F2-programmed SHR male offspring (blue patterned bars), F2-programmed SHR-Zbtb16 male offspring (green patterned bars) in 6 months of age. The strain comparison using the post-hoc Fisher’s least significant difference test of the two-way ANOVA for STRAIN and PROGRAM as major factors are indicated as follows: * *p* < 0.05, ** *p* < 0.01, *** *p* < 0.001. Effect of programming in SHR and SHR-Zbtb16 strains is represented by blue and green asterisks (*), respectively. (**a**,**c**) C represents STRAIN differences between F2 SHR and SHR-Zbtb16 control males, C15 *, C16 **, C17 *, TG10 *, TG15 *, which were all higher in SHR-Zbtb16 F2 control males.

We observed similar profiles of cholesterol and triacylglycerols distribution into lipoprotein fractions of both strains of offspring. There were minor differences in very large to medium HDL cholesterol levels, which were higher in control SHR-Zbtb16 group, the same was true for small LDL and very large HDL triacylglycerols (Figure 9). Most prominent effect of second-generation HSD - programming was a significant increase in medium to very small HDL cholesterol in both strains (Figure 9a,b), with the additional increase of very large and large HDL particle levels in SHR-Zbtb16. Concurrently with the increase of HDL cholesterol, the size of HDL particles had decreased significantly (HDL cholesterol particle size; 12.28 ± 0.06 nm (SHR F2 control males) vs. 11.93 ± 0.06 nm (SHR F2 programmed males), *p* = 0.002; 12.35 ± 0.05 nm (SHR-Zbtb16 F2 control males) vs. 11.94 ± 0.08 nm (SHR-Zbtb16 F2 programmed males), *p* = 0.0004). Triacylglycerol levels were lower in small to very small LDL and very large to medium HDL in F2 programmed offspring compared to control groups, again with a more prominent effect in SHR-Zbtb16 (Figure 9c,d).

## 4. Discussion

We present data on how sucrose feeding in pregnancy and lactation of F0 generation rat dams affect metabolic profiles of standard-diet-fed F1 and F2 offspring generations. Particularly in F1, these effects could be, in part, predictive adaptive responses (PARs), which are environmental responses without an immediate benefit to the organism, but rather a distant benefit, e.g., the ability to anticipate the future environment [28] and adapt. PARs presumably evolved to enable organisms to cope with transient changes in the environment and therefore “provide a process by which individuals adapt to their future postnatal environment by restricting their range of possible phenotypes to a narrower spectrum, without changing the genotype [28]”.

In our study, we observed strain-specific body weight differences in the F1-programmed generation of females prior to pregnancy, during which the distinction disappeared. Control and F1-programmed females also differed in their body weight, as the programming by maternal HSD contributed to lower body weight of F1 groups compared to controls and this difference persisted up to the first week of pregnancy. Body weight of the lactating rat dams did not significantly differ, as opposed to the group of their F0 HSD-fed mothers, whose weight dropped significantly in this period [8]. The fasting glycaemia and insulin levels were increased in F1-programmed females in adulthood, which suggests a possible inclination to impaired glucose tolerance. The effect of pregnancy on glucose tolerance was comparable between both groups and strains, although SHR F1-programmed pregnant dams showed an additional decrease of blood glucose levels halfway through OGTT (t = 90 min). Interestingly, HSD programming seemed to alter the appetite of F1-programmed females, as they consumed more of the chow during pregnancy than their control group, which slightly increased their energy intake. This is consistent with previous studies which had shown that sucrose diet induced elevated food intake and appetite in offspring exposed to maternal diet containing fructose, either bound (sucrose) or free fructose in form of high fructose corn syrup [29,30]. Effects of maternal (F0) HSD feeding in F1 programmed males are a part of different project and therefore not discussed here.

Six-month-old SHR F2-programmed males showed lower fasting glycaemia compared to controls, as did SHR F1-programmed males [8]. We observed that the effect of HSD-programming of their mothers acted differently upon genomic background, as SHR-Zbtb16 F2-programmed males showed no difference in fasting glycaemia, but inclined to biphasic course of OGTT [31], with a significant drop of blood glucose levels at t = 60 min with which they differed not only from their controls but also SHR F2-programmed males. Fasting insulin levels of F2-programmed males were elevated compared to their controls in a similar fashion as in their F1-programmed mothers, although only SHR-Zbtb16 groups’ increase was statistically significant. Decrease of small to very small LDL triacylglycerols in F2-programmed groups, with more prominent effect in SHR-Zbtb16 was similar as it was in F1-programmed males [8], who directly interacted with HSD through the uteroplacental system and breast milk of F0 mothers. The persistence of this specific decrease seems to be determined by the HSD effect even in second generation of offspring. A similar pattern was observed in decrease of medium HDL triacylglycerols which was more prominent in SHR-Zbtb16 [8] F1-programmed males. In addition, we observed a decrease of very large HDL triacylglycerols in F2-programmed males. Interestingly, medium to very small HDL cholesterol levels have been increased significantly only in F2-programmed males, together with a decrease of size of these particles.

The maternal HSD-specific milieu possibly programmed the offspring for the environment providing high amounts of sucrose, however in adult life their main source of carbohydrates from STD was starch. Predictive adaptive hypothesis postulates that environmental mismatch of early life versus adulthood can increase the risk of disease [32,33]. However, historically, it was poor maternal nutrition in early development versus overnutrition in adulthood due to westernized diet popularity that was the most studied model of disparity. Our study established the opposite conditions, whereby maternal diet of F0 generation abundant in sucrose, although the same in calorie content as STD, is followed by STD consumption after weaning of their F1 offspring and whole pre- and postnatal life of their grandsons. If the strategies for maximizing the postnatal survival success are based on the anticipation of a particular adult environment, it is possible that the metabolic systems of F1 and F2 offspring were prepared to manage increased levels of sucrose and thus overproduction of triglycerides and responded with a higher baseline for HDL production in order to alleviate these effects. Triglycerides are being transferred from VLDL to HDL by the action of cholesterol ester transfer protein [34]. After hydrolysis by hepatic lipase they are cleared from plasma, which serves as a basis for protective effect of HDL against dyslipidemia and coronary heart disease. However, a more comprehensive design, including all matched (including re-exposure to sucrose group) and mismatched pre- and postnatal environments, would be necessary to substantiate the above mechanism. 

Another effect of HSD-programming in early life that persisted two generations of offspring was a significant increase of interscapular brown fat weight. We already noticed this effect in F1-programmed adult males of both strains [8], although direct programming with HSD resulted in higher increase in F1-programmed males than the increase we observed in second generation. As shown lately, brown fat represents an essential regulator of the effect of maternal nutritional programming [35]. Body fat distribution in terms of retroperitoneal fat and epididymal fat, partly correlating with human visceral fat [36], wasn’t affected by the second-generation programming. Relative weights of retroperitoneal fat differed among the strains in F2 generation of programmed males, with SHR having higher relative weights. Strains used in this study differed in a single gene mutation—254 kb deletion in intronic region of *Zbtb16* gene of the PD/Cub strain origin [37]. The genomic background of SHR was thought to exacerbate the effects of Zbtb16 gene involved in pathogenesis of metabolic syndrome [18], but conversely was shown to improve the related parameters [20], which was to an extent apparent in our study as well.

We are aware of several inherent limitations of this study. First, we studied only the male offspring of two specific, inbred rat strains within a single protocol of exposition to HSD. This mostly followed our previous studies, maximizing the homogeneity of the control and experimental groups in order to observe the subtle effects due to intergenerational transfer [8,38]. Also, the obtained results do not provide a direct mechanistic link between *Zbtb16* and the observed metabolic changes. Thus, comprehensive genetic and epigenetic studies will be necessary to dissect these aspects in detail. In conclusion, we show the second-generation effects of maternal exposition to a high-sucrose diet, some modulated to a certain extent by variation in the *Zbtb16* gene. 

## Figures and Tables

**Table 1:** Areas under glycaemic curves (AUC) values calculated from course of oral glucose tolerance tests in SHR and SHR-Zbtb16 mothers and their male offspring according to Heikkinen et al. [27].

	AUC (mmol/L/180 min), mean ± SEM	
	Adulthood	Pregnancy
**F1 mothers**		
SHR F1 control	587 ± 76	347 ± 44
SHR-Zbtb16 F1 control	383 ± 42	211 ± 43
SHR F1 programmed	385 ± 38	280 ± 32
SHR-Zbtb16 F1 programmed	345 ± 42	244 ± 36
**F2 male offspring**		
SHR F2 control	372 ± 32	
SHR-Zbtb16 F2 control	458 ± 19	
SHR F2 programmed	379 ± 16	
SHR-Zbtb16 F2 programmed	356 ± 21	
